# Supplementary material for: Clinical and molecular correlation defines activity of physiological pathways in life-sustaining kidney xenotransplantation
Source: Nat Commun. 2023 Jun 13;14:3022. doi: 10.1038/s41467-023-38465-x (PMC10264453; doi:10.1038/s41467-023-38465-x)
Supplement: Supplementary file 1 — Supplementary Information [file 41467_2023_38465_MOESM1_ESM.pdf]

Supplementary Information for:

Title: Clinical and molecular correlation defines activity of physiological pathways in life-sustaining kidney xenotransplantation

Authors: Daniel J Firl[1,2#], Grace Lassiter[1,#], Takayuki Hirose[1], Robert Policastro[2], Ashley D'Attilio[1], James F Markmann[1], Tatsuo Kawai[1,§], Katherine C Hall[2,§,*]

Affiliations:
[1]Center for Transplantation Sciences, Massachusetts General Hospital, Boston, MA
[2]eGenesis Inc, Cambridge, MA
[#]These authors contributed equally
[§]These authors jointly supervised the work

*Corresponding author:
Katherine C Hall, PhD
eGenesis, Inc
101 Cambridgepark Drive, Suite 200
Cambridge, MA
Katherine.hall@egenesisbio.com

Running title (35characters including spaces): Physiology of pig to NHP kidney xenotransplant

Keywords: xenotransplantation, physiology of xenotransplantation, porcine kidney physiology

Supplementary Table 1: Recipient survival, sex, age, weight, and data collection by animal

Animal ID	Survival (days)	Sex	Age[b] (years)	Weight[b] (kg)	CC	UA	EP	CTx	PRA	Ald	US	RNA-seq
M2420	>648	Male	8.0	7.96	X	X	X		X		X	X
M2519	511	Male	8.0	6.26	X	X	X		X		X	X
M7519	243	Male	5.2	6.81	X		X	X	X		X	X
M8020	266	Female	6.1	4.57	X		X	X	X		X	X
M6120	240	Male	4.8	6.30	X		X	X			X	
M7920	64	Female	5.5	4.27	X						X	X
M8220[a]	103	Female	8.5	4.46	X		X	X			X	X
M8320	>451	Female	8.4	4.40	X	X	X				X	X
M4321[a]	119	Male	9.1	7.61	X			X	X	X	X	
M5821	205	Male	8.5	8.72	X			X		X	X	
M6521[a]	176	Male	6.6	7.04	X		X		X	X	X	X
M6121	283	Male	7.3	7.07	X	X	X	X		X	X	X
M7721	>263	Male	4.6	4.80	X	X				X	X	
M7621	>207	Male	5.5	4.94	X	X	X			X	X	
M1322[a]	82	Male	6.1	6.69	X	X					X	
M2021	>95	Male	5.1	5.05	X	X					X	
M1522	90	Male	5.0	5.96	X	X					X	

[a]Denotes delayed native nephrectomy for single native kidney left at time of transplantation, [b]Values at the time of transplant

Abbreviations: Ald = aldosterone, CC = clinical chemistry, CTx = beta-C-terminal telopeptide, EP = endocrine panel (calcifediol, calcitriol, PTH, PTHrP), ID = identification, PRA = plasma renin activity, UA = urinalysis, US = ultrasonography

Supplementary Table 2: Genetic composition, age and weight of gene edited Yucatan minipig donors

Genotype	Recipient ID	KO	KI - CRPs	KI - TRPs	KI - IRPs	Age (months)	Weight (kg)
EGEN2734	M2420	3KO	*CD46, CD55*	*PROCR, THBD*	*HMOX1, TNFAIP3, CD47*	2.3	9.6
EGEN2734	M2519	3KO	*CD46, CD55*	*PROCR, THBD*	*HMOX1, TNFAIP3, CD47*	2.5	11.0
EGEN2060	M7519	3KO	*CD46, CD55*	*TFPI, THBD*	*B2M/HLA-E, CD47*	1.9	11.6
EGEN2060	M8020	3KO	*CD46, CD55*	*TFPI, THBD*	*B2M/HLA-E, CD47*	2.1	6.3
EGEN2734	M6120	3KO	*CD46, CD55*	*PROCR, THBD*	*HMOX1, TNFAIP3, CD47*	2.4	11.8
EGEN2734	M7920	3KO	*CD46, CD55*	*PROCR, THBD*	*HMOX1, TNFAIP3, CD47*	3.1	19.5
EGEN2734	M8220[a]	3KO	*CD46, CD55*	*PROCR, THBD*	*HMOX1, TNFAIP3, CD47*	3.8	19.0
EGEN2734	M8320	3KO	*CD46, CD55*	*PROCR, THBD*	*HMOX1, TNFAIP3, CD47*	3.1	16.8
EGEN2574	M4321[a]	3KO	*CD46, CD55*	*TFPI, PROCR, THBD*	*B2M/HLA-E, CD47*	2.1	10.4
EGEN2574	M5821	3KO	*CD46, CD55*	*TFPI, PROCR, THBD*	*B2M/HLA-E, CD47*	2.4	13.5
EGEN2784	M6521[a]	3KO, RI	*CD46, CD55*	*PROCR, THBD*	*HMOX1, TNFAIP3, CD47*	3.3	17.8
EGEN2784	M6121	3KO, RI	*CD46, CD55*	*PROCR, THBD*	*HMOX1, TNFAIP3, CD47*	4.2	27.0
EGEN2734	M7721	3KO	*CD46, CD55*	*PROCR, THBD*	*HMOX1, TNFAIP3, CD47*	1.2	6.4
EGEN2784	M7621	3KO, RI	*CD46, CD55*	*PROCR, THBD*	*HMOX1, TNFAIP3, CD47*	1.9	10.0
EGEN2060	M1322[a]	3KO	*CD46, CD55*	*TFPI, THBD*	*B2M/HLA-E, CD47*	2.6	11.4
EGEN2060	M2021	3KO	*CD46, CD55*	*TFPI, THBD*	*B2M/HLA-E, CD47*	2.4	10.8
EGEN2060	M1522	3KO	*CD46, CD55*	*TFPI, THBD*	*B2M/HLA-E, CD47*	2.4	10.8

[a]Denotes delayed native nephrectomy for single native kidney left at time of transplantation

Abbreviations: KOs = knockouts, 3KO = knockouts for GGTA1/CMAH/B4GALNT2, *B2M/HLA-E* = gene name reflecting a fusion construct leading to expression of the protein beta-2-microglobulin and human leukocyte antigen-E , *CD46* = gene name for the protein, cluster of differentiation 46, *CD47* = gene name for the protein, cluster of differentiation 47, *CD55* = gene name for the protein, cluster of differentiation 55, CRPs = complement regulatory proteins, *HMOX1* = gene name for the protein, heme oxygenase 1, KI = knockin of human transgenes, IRPs = inflammatory and immunity regulating proteins, *PROCR* = gene name for the protein, endothelial cell protein C receptor (EPCR), RI = retroviral inactivation, *TFPI* = gene name for the protein, tissue factor pathway inhibitor, *THBD* = gene name for the protein, thrombomodulin , *TNFAIP3* = gene name for the protein, tumor necrosis factor alpha-induced protein 3 also known as A20, TRPs = thromboregulatory proteins

Supplementary Table 3: Summary findings of hypercalcemia, hypophosphatemia and hydronephrosis

Animal ID	Survival (days)	Hypercalcemia	Hypophosphatemia	Early Post-Transplantation Hydronephrosis[#]
M7920	64	+	+	Yes, SFU-II
M1322[a]	82	+	+	No
M1522	90	+	+	Yes, SFU-III
M8220[a]	103	+	+	No
M4321[a]	119	+	+	No
M6521[a]	176	+	+	Yes, SFU-I
M5821	205	+	+	Yes, SFU-II
M6120	240	+	+	No
M7519	243	+	+	Yes, SFU-II
M8020	266	+	+	No
M6121	283	+	+	Yes, SFU-II
M2519	511	+	+	Yes, SFU-III
M2021	>95	+	+	Yes, SFU-II
M7621	>207	+	+	Yes, SFU-IV
M7721	>263	+	+	Yes, SFU-III
M8320	>451	+	+	Yes, SFU-II
M2420	>648	+	+	No

[a]Denotes delayed native nephrectomy for single native kidney left at time of transplantation
[#]Denotes hydronephrosis graded within the first 30days PTT
Abbreviations: CC = clinical chemistry, EP = endocrine panel, PRA = plasma renin activity, SFU = society for fetal urology, US = ultrasonography

Supplementary Table 4: Generalized linear modeling of absolute xenograft growth over time

	Estimate (cm)	Std. Error	t-value	*P*-value
Intercept	8.33	0.19	43.59	<2E-16
Post-transplant time (days)	0.0014	0.0004	3.72	2.3E-4
Factor(animalid)				
M1522	0.43	0.28	1.55	0.123
M2021	-0.31	0.27	-1.15	0.251
M2420	-0.81	0.22	-3.63	0.0003
M2519	-0.87	0.22	-3.90	0.0001
M4321	-0.74	0.28	-2.66	0.0081
M5821	0.79	0.25	3.19	0.0016
M6120	-0.17	0.25	-0.69	0.494
M6121	-0.02	0.24	-0.09	0.928
M6521	-1.08	0.30	-4.30	2.12E-5
M7519	0.05	0.27	0.19	0.847
M7621	0.02	0.31	0.07	0.948
M7721	-1.19	0.20	-4.48	9.74E-6
M7920	-0.34	0.25	1.76	0.266
M8020	-0.17	0.23	-0.72	0.474
M8220	-0.31	0.27	-1.12	0.263
M8320	-1.25	0.23	-5.32	1.74E-7
Null deviance: 352.87 on 422 degrees of freedom<br>Residual deviance: 234.63 on 405 degrees of freedom<br>AIC: 989.12				

Supplementary Table 5: Generalized linear modeling of relative xenograft growth over time

		Estimate (%)	Std. Error	t-value	*P*-value
Intercept		7.29	2.45	2.97	0.0032
Post-transplant time (days)		0.022	0.0047	4.77	2.62E-6
Factor(animalid)					
M1522		-2.42	3.59	-0.67	0.502
M2021		-13.02	3.52	-3.70	2.5E-4
M2420		-16.01	2.84	-5.63	3.5E-8
M2519		-28.31	2.84	-9.97	<2E-16
M4321		16.00	3.60	4.45	1.1E-5
M5821		-8.51	3.16	-2.69	0.0074
M6120		-17.11	3.22	-5.31	1.9E-7
M6121		-25.29	3.02	-8.38	1.1E-15
M6521		-29.62	3.19	-9.30	<2E-16
M7519		-11.43	3.02	-3.78	1.8E-4
M7621		7.19	3.68	1.95	0.052
M7721		-9.80	3.40	-2.88	0.004
M7920		5.14	4.05	1.27	0.206
M8020		-0.62	2.96	-0.21	0.834
M8220		3.06	3.40	0.90	0.368
M8320		-15.78	2.97	-5.31	1.9E-7
Null deviance: 80615 on 392 degrees of freedom					
Residual deviance: 31312 on 375 degrees of freedom					
(30 observations deleted due to missingness)					
AIC: 2873.8					

Supplementary Figures:

Supplementary Figure 1: Longitudinal presentation of RAAS activity measurements

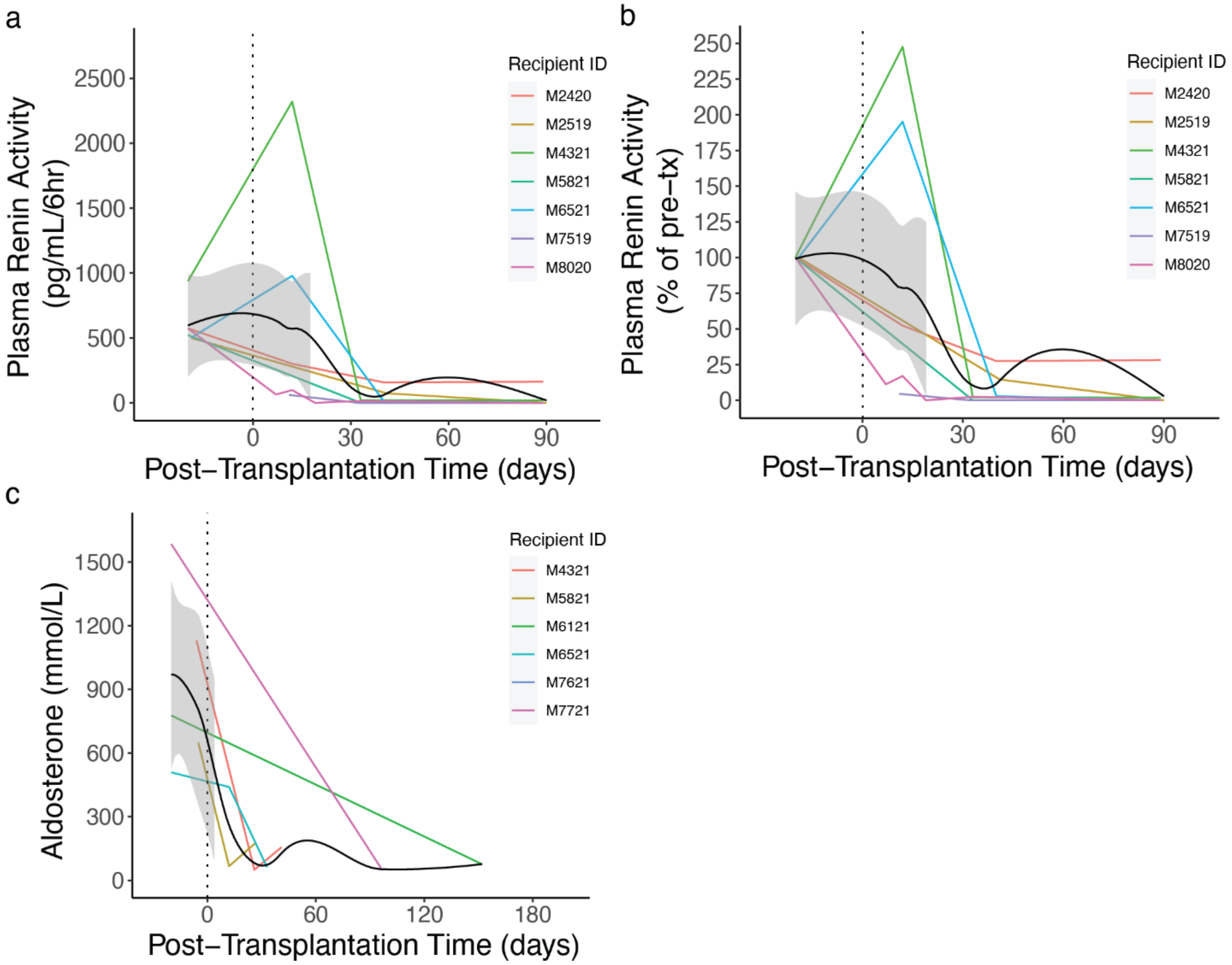

Supplementary Figure 1: Longitudinal analysis of RAAS components mirrors binned data. a) Plasma renin activity as measured in NHPs pre-transplantation and post-transplantation longitudinally demonstrates decreased PRA after transplantation. Note that animals M5821 and M6521 had delayed native nephrectomy and increase in PRA after transplantation were during the interval where the animals had a single NHP kidney in place. Black solid line is the LOESS estimate, a non-parametric regression method estimating the relationship between creatinine and PTT across all studies; grey shaded region is 95% confidence interval for the estimate. Dotted line at PTT 0 represents transition from pre- to post-transplant period. b) As in a) but for percent activity instead of absolute activity. c) As in a but for measurement of Aldosterone, one of the end products of the RAAS pathway. n=7 biologically independent transplants for panels a – b and

n=6 biologically independent transplants for panel c. Abbreviations: ID = identifier, LOESS = locally estimated scatterplot smoothing, hr = hours, NHPs = non-human-primates, pre-tx = pre-transplant, PTT = post-transplantation time.

Supplementary Figure 2: Study design focusing on immunosuppressive regiment for allotransplanted NHPs

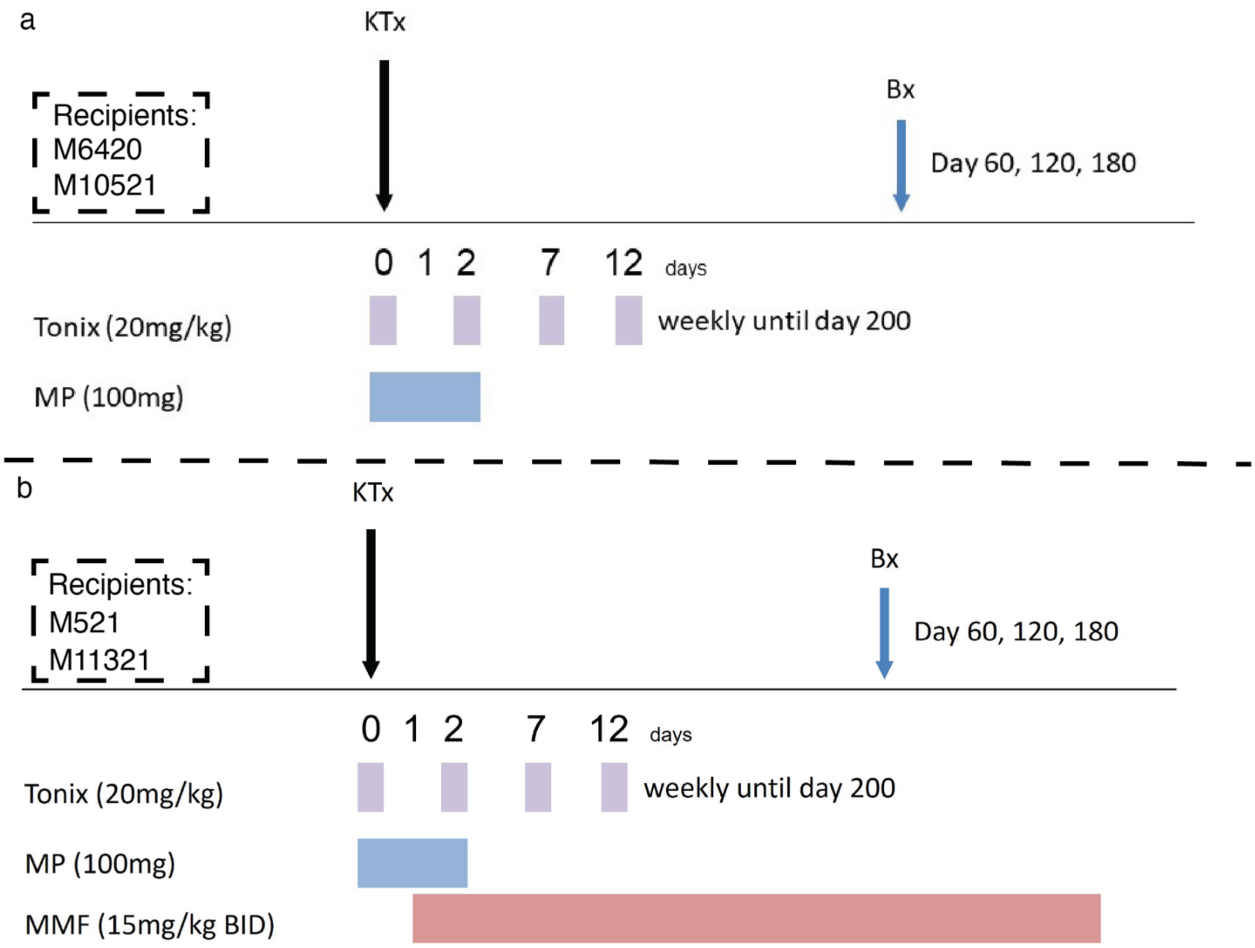

Supplementary Figure 2: Schematic outlining the study design and especially the immunosuppressive regimen administered to allotransplanted NHPs. a) Immunosuppressive regimen including steroids (quickly tapered) and anti-CD154 antibody only. b) Immunosuppressive regimen including steroids (quickly tapered), mycophenylate mofetil alongside anti-CD154 antibody. Anti-CD154 antibody (TNX-1500) was weaned from day 200 onward until frank clinical rejection in both a and b. Abbreviations: Bx = allograft biopsy, KTx = kidney allotransplantation, MMF = mycophenylate mofetil, MP = methylprednisolone, NHP = non-human-primates, Tonix = TNX-1500 anti-CD154 antibody

Supplementary Figure 3: Serum renin activity (SRA) in NHPs after allo- compared to xeno-transplantation

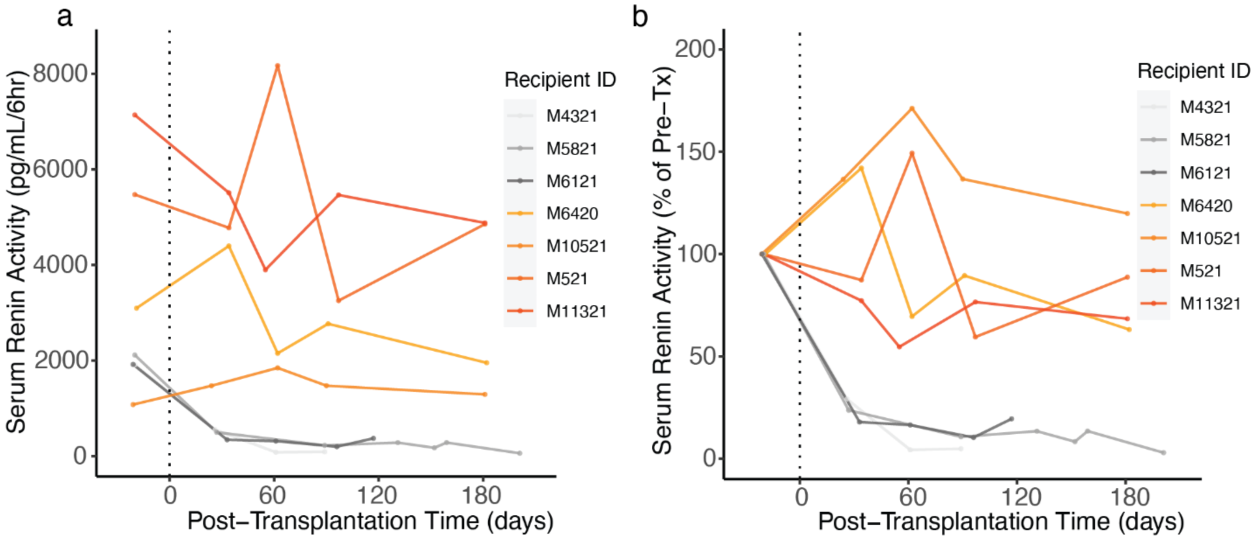

Supplementary Figure 3: a) Longitudinal analysis of absolute serum renin activity for NHPs after allotransplantation (oranges) vs xenotransplantation (grays). Dotted line at PTT 0 represents transition from pre- to post-transplant period. b) As in a) but for relative SRA comparison. n=7 biologically independent transplants for panels a – b. Abbreviations: hr = hours, ID = identifier, NHPs = non-human-primates, Pre-Tx = pre-transplantation, PTT = post-transplantation time.

Supplementary Figure 4: Additional clinical chemistry data for NHPs after transplantation

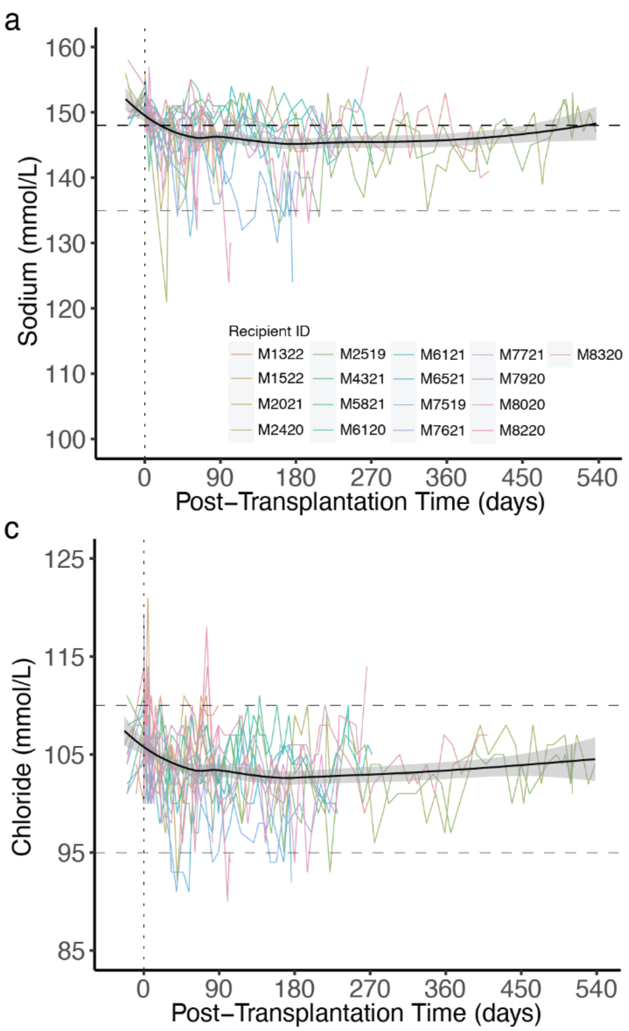

Supplementary Figure 4: Longitudinal analysis of clinical chemistries demonstrates most analytes are in a normal range post-transplantation. Clinical chemistries were monitored 1-2x per week for alterations in a) sodium, b) potassium, and c) chloride. d) Additional data points were generated measuring the ionized calcium which confirms observations of hypercalcemia in a protein-independent manner. For panels a – d, black solid line is the LOESS estimate, a non-parametric regression method estimating the relationship between each analyte and PTT across all studies; grey shaded region is 95% confidence interval for the estimate. Dotted line at PTT 0 represents transition from pre- to post-transplant period. Dashed lines represent normal range as provided by the Center for Comparative Medicine from MGH. n=17 biologically independent transplants for panels a – c. n=13 biologically independent transplants for panel d. Abbreviations: LOESS = locally estimated scatterplot smoothing, MGH = Massachusetts General Hospital, PTT = post-transplantation time.

Supplementary Figure 5: Clinical chemistry data in NHPs after allo- compared to xeno-transplantation

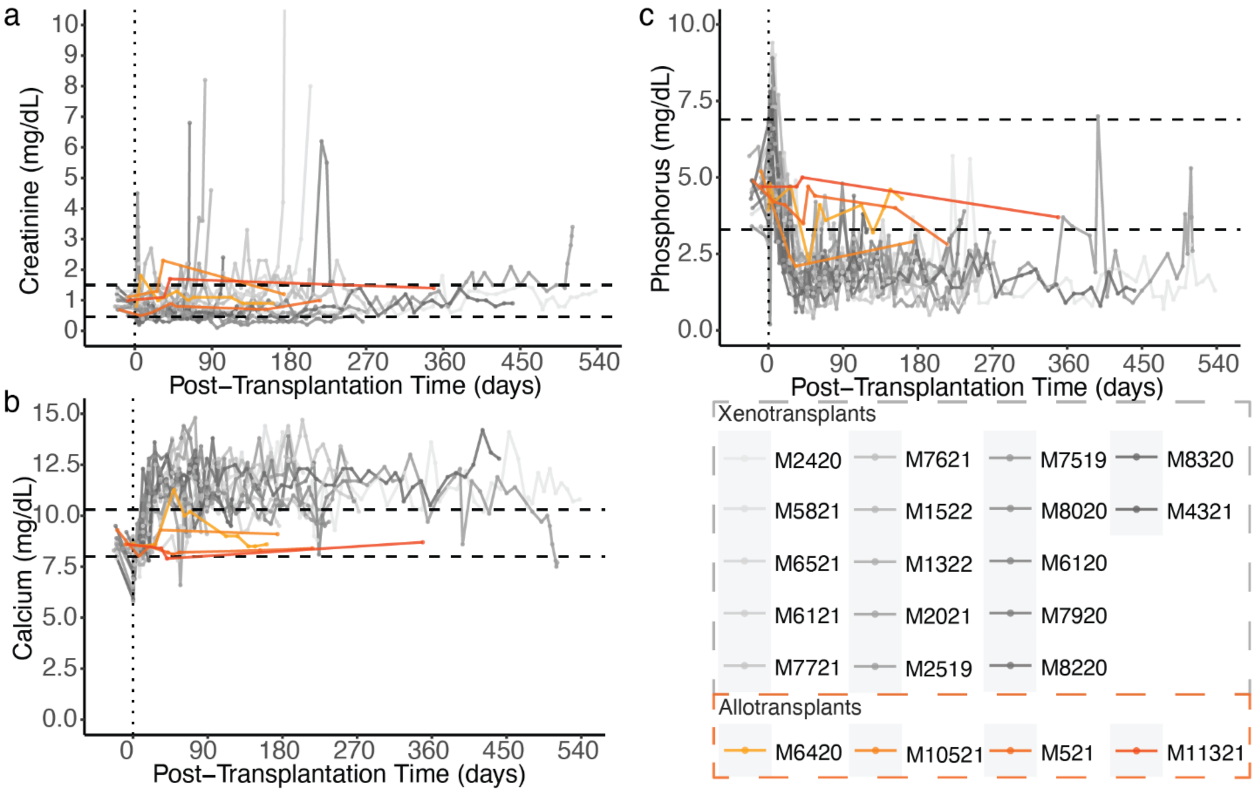

Supplementary Figure 5: Longitudinal analysis of clinical chemistries for NHPs after allotransplantation (oranges) vs xenotransplantation (grays). Clinical chemistries were monitored 1-2x per week for alterations in serum: a) creatinine, b) calcium, and c) phosphorus. n=21 biologically independent transplants for panels a – c. Abbreviations: NHPs = non-human-primates.

Supplementary Figure 6: Additional urine data for NHPs after transplantation

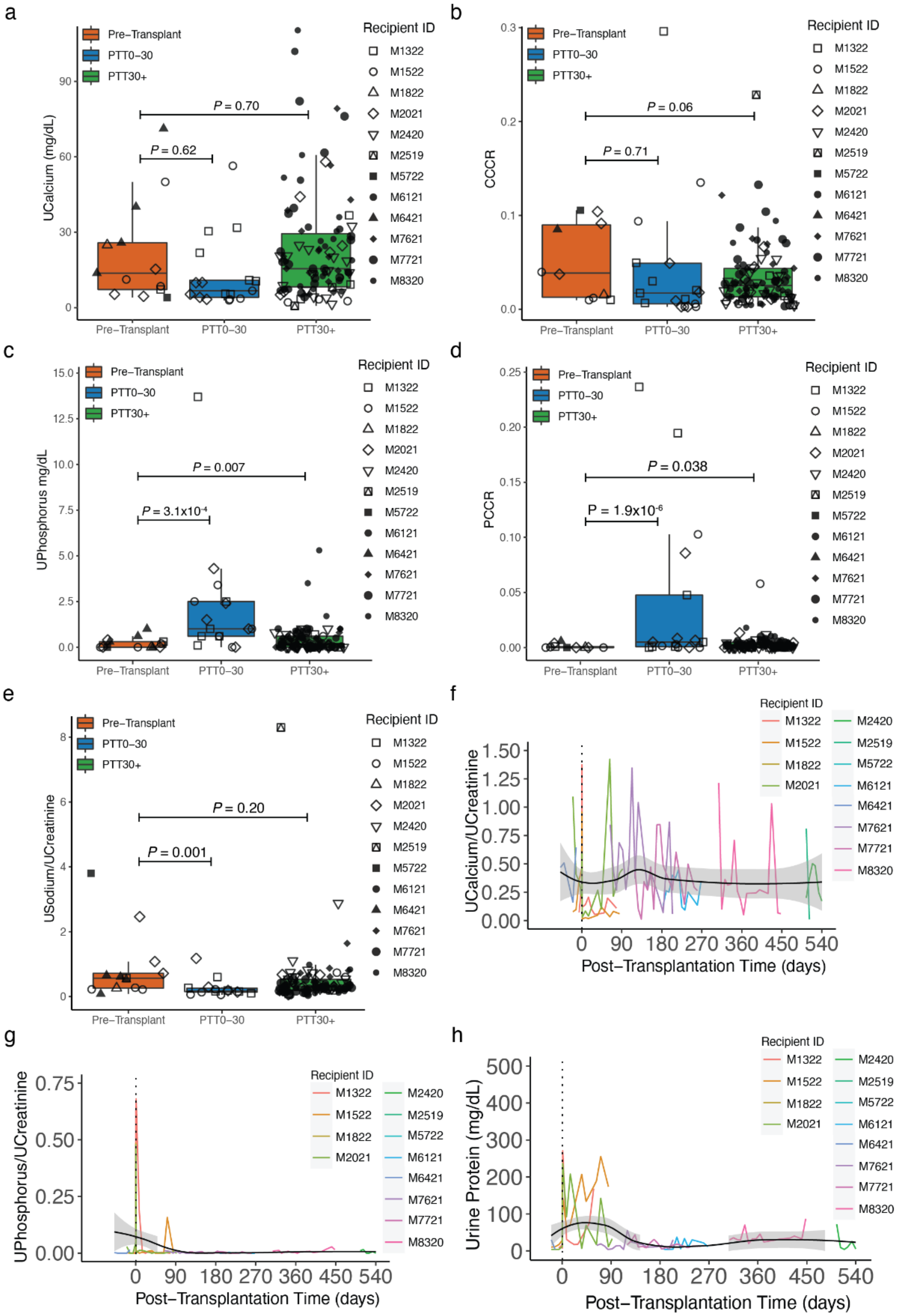

Supplementary Figure 6: Additional urinalysis data from long-term survivors demonstrates similar trends as in simple urine studies normalized to urinary creatinine. a) Binned urine measurements for non-normalized measurements of urinary

calcium and b) urinary calcium to creatinine clearance ratio. c) As in a) but for urinary phosphorus. d) As in b) but for urinary phosphorus to creatinine clearance ratio. e) Binned urine measurements for urinary sodium to creatinine ratio. f) Longitudinal urine calcium to creatinine ratio. g) As in f) but for urinary phosphorus to creatinine ratio. h) Urinary protein measurements longitudinally. For panels f – h, black solid line is the LOESS estimate, a non-parametric regression method estimating the relationship between each analyte and PTT across all studies; grey shaded region is 95% confidence interval for the estimate. Dotted line at PTT 0 represents transition from pre- to post-transplant period. For box and whisker plots, the central line in each box represents the median of the distribution, i.e., the value that separates the lower 50% of observations from the upper 50%. The box itself represents the interquartile range (IQR), which spans from the 25th to the 75th percentile of the distribution. The lower bound of the box is the first quartile (Q1), while the upper bound is the third quartile (Q3). The whiskers of the box and whisker plot extend from the box to the minimum and maximum observations within 1.5 times the IQR of the lower and upper quartile, respectively (panels c and d). Statistical testing for panels a – e was performed using generalized linear models with xenotransplantation bin (pre vs post) and recipient IDs as factor variables. n=12 biologically independent transplants for panels a – h. Abbreviations: CCCR = calcium to creatinine clearance ratio, ID = identifier, IQR = inter-quartile range (25th – 75th percentiles), LOESS = locally estimated scatterplot smoothing, PCCR = phosphorus to creatinine clearance ratio, PTT = post-transplantation time, UCalcium = urinary calcium, UCreatinine = urinary creatinine, UPhosphorus = urinary phosphorus.

Supplementary Figure 7: Analysis of selected genes from bulk RNAseq for calcium/phosphorus pathways

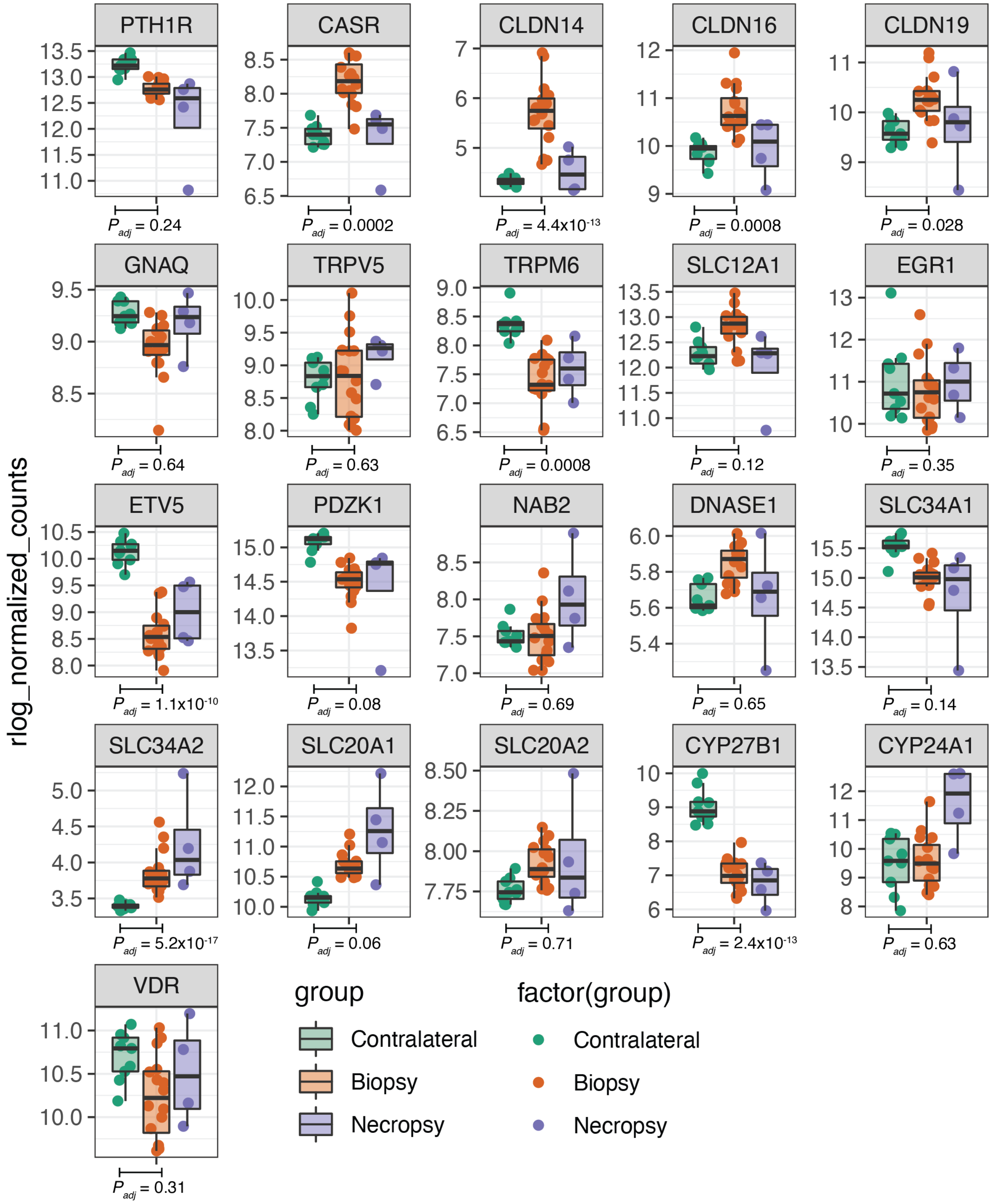

Supplementary Figure 7: Quantitative analysis of porcine RNAseq data for specific genes related to calcium and phosphorus handling. A series of genes related to calcium and phosphorus handling were queried for expression amongst samples taken

from contralateral untransplanted kidneys (light green), biopsy samples (light orange), and necropsy samples (light purple). The central line in each box represents the median of the distribution, i.e., the value that separates the lower 50% of observations from the upper 50%. The box itself represents the interquartile range (IQR), which spans from the 25th to the 75th percentile of the distribution. The lower bound of the box is the first quartile (Q1), while the upper bound is the third quartile (Q3). The whiskers of the box and whisker plot extend from the box to the minimum and maximum observations within 1.5 times the IQR of the lower and upper quartile, respectively. Statistical comparisons between contralateral and biopsy tissue (no consideration of necropsy samples) are provided by DESeq2 (methods), adjusted for multiplicity of testing using the FDR method described by Benjamini-Hochberg, adjusted P values (P_{adj}) are provided below each gene plot. (Groupwise sample composition is contralateral untransplanted kidney, n=9 biologically independent samples, biopsy kidney tissue, n=16 biologically independent samples, and necropsy kidney tissue, n=4 biologically independent samples.

Supplementary Figure 8: Longitudinal presentation of calcium-vitamin D-parathyroid hormone axis activity

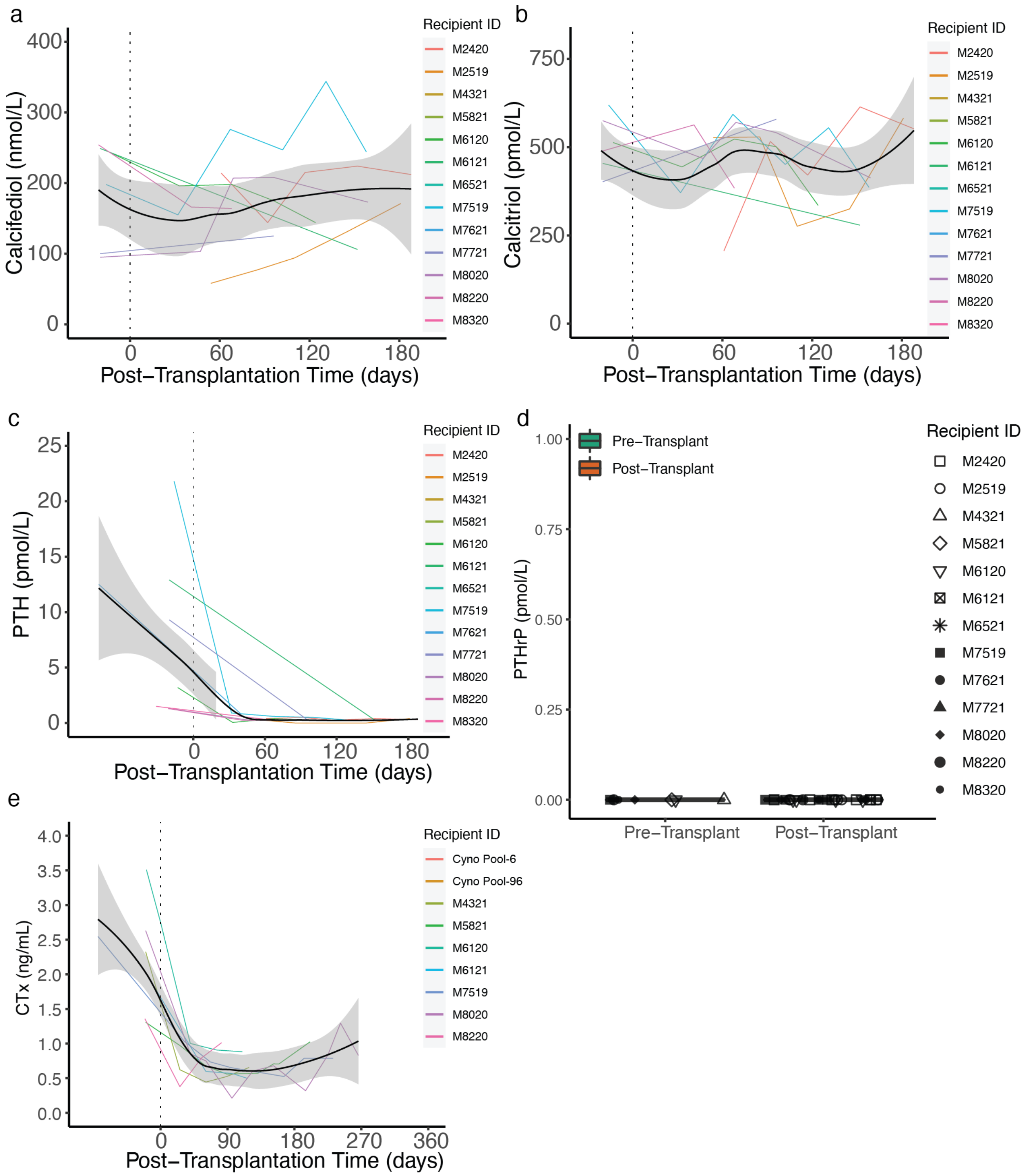

Supplementary Figure 8: Longitudinal analysis of calcium-PTH-vitamin D components mirrors binned data. a) Calcifediol as measured in cynomolgus macaques pre-transplantation and post-transplantation longitudinally demonstrates no significant changes after transplantation. b) As in a) but for Calcitriol. c) As in a but for measurement of PTH which demonstrates rapid and significant decreases after transplantation in the presence of hypercalcemia. d) Binned PTHrP data with no detectable

PTHrP at any timepoint for any study. e) As in a but for measurement of CTx which is significantly decreased after transplantation in the presence of hypo-PTH and hypercalcemia. For panels a, b, c, e, black solid line is the LOESS estimate, a non-parametric regression method estimating the relationship between each analyte and PTT across all studies; grey shaded region is 95% confidence interval for the estimate. Dotted line at PTT 0 represents transition from pre- to post-transplant period. Box and whisker plots of distributions are drawn with a central bar at the median, a box around the IQR with lower bound 25th percentile and upper bound 75th percentile and whiskers drawn to length 1.5x the IQR, ie, the minima and maxima of the whiskers, all data points are drawn over the box and whisker plots (panel d). n=13 biologically independent transplants for panels a – e. Abbreviations: CTx = beta-C-terminal telopeptide, Cyno6 = pool of 6 cynomolgus macaques' serum (not transplanted), Cyno96 = pool of 96 cynomolgus macaques' serum (not transplanted), ID = identifier, IQR = inter-quartile range (25th – 75th percentiles), LOESS = locally estimated scatterplot smoothing, PTH = parathyroid hormone, PTT = post-transplantation time, PTHrP = parathyroid hormone related peptide.
