# Supplementary material for: Clinical and molecular correlation defines activity of physiological pathways in life-sustaining kidney xenotransplantation
Source: Nat Commun. 2023 Jun 13;14:3022. doi: 10.1038/s41467-023-38465-x (PMC10264453; doi:10.1038/s41467-023-38465-x)
Supplement: Supplementary file 3 — Description of Additional Supplementary Files [file 41467_2023_38465_MOESM3_ESM.pdf]

Description of Additional Supplementary Files

Supplementary Data 1: Gene edited porcine donor chemistries
