# Supplementary material for: Clinical and molecular correlation defines activity of physiological pathways in life-sustaining kidney xenotransplantation
Source: Nat Commun. 2023 Jun 13;14:3022. doi: 10.1038/s41467-023-38465-x (PMC10264453; doi:10.1038/s41467-023-38465-x)
Supplement: Supplementary file 5 — Reporting Summary [file 41467_2023_38465_MOESM5_ESM.pdf]

nature portfolio

Corresponding author(s): Katherine Hall

Last updated by author(s): 2 May 2023

Reporting Summary

Nature Portfolio wishes to improve the reproducibility of the work that we publish. This form provides structure for consistency and transparency in reporting. For further information on Nature Portfolio policies, see our Editorial Policies and the Editorial Policy Checklist.

Statistics

For all statistical analyses, confirm that the following items are present in the figure legend, table legend, main text, or Methods section.

n/a	Confirmed	
☐	☒	The exact sample size (*n*) for each experimental group/condition, given as a discrete number and unit of measurement
☐	☒	A statement on whether measurements were taken from distinct samples or whether the same sample was measured repeatedly
☐	☒	The statistical test(s) used AND whether they are one- or two-sided<br>*Only common tests should be described solely by name; describe more complex techniques in the Methods section.*
☐	☒	A description of all covariates tested
☐	☒	A description of any assumptions or corrections, such as tests of normality and adjustment for multiple comparisons
☐	☒	A full description of the statistical parameters including central tendency (e.g. means) or other basic estimates (e.g. regression coefficient) AND variation (e.g. standard deviation) or associated estimates of uncertainty (e.g. confidence intervals)
☐	☒	For null hypothesis testing, the test statistic (e.g. *F*, *t*, *r*) with confidence intervals, effect sizes, degrees of freedom and *P* value noted<br>*Give P values as exact values whenever suitable.*
☒	☐	For Bayesian analysis, information on the choice of priors and Markov chain Monte Carlo settings
☒	☐	For hierarchical and complex designs, identification of the appropriate level for tests and full reporting of outcomes
☒	☐	Estimates of effect sizes (e.g. Cohen's *d*, Pearson's *r*), indicating how they were calculated

Our web collection on statistics for biologists contains articles on many of the points above.

Software and code

Policy information about availability of computer code

Data collection	For all devices or machines used in data collection, the commercial software provided by the manufacturer of the device was used. No additional specialty or custom software was used.
Data analysis	The following libraries were used in RStudio (2022.02.2 Build 485) with R (4.2.0): tximport (1.22.0), DESeq2 (1.36.0), ComplexHeatmap (2.12.1), pathfindR (1.6.4), DropletUtils (1.14.2), scDblFinder (1.8.0), bluster (1.4.0), fgsea (1.20.0), ggplot2(3.2.0), scran (1.22.1), Seurat (4.1.0), scater (1.22) and gmless (5.4.12). The following programs were run on the command line: gffread (0.12.7), salmon (1.6.0), and STARsolo (2.7.9a). scvi (0.15.5) was run in Python (3.8.10).

For manuscripts utilizing custom algorithms or software that are central to the research but not yet described in published literature, software must be made available to editors and reviewers. We strongly encourage code deposition in a community repository (e.g. GitHub). See the Nature Portfolio guidelines for submitting code & software for further information.

Data

Policy information about availability of data

All manuscripts must include a data availability statement. This statement should provide the following information, where applicable:

- Accession codes, unique identifiers, or web links for publicly available datasets
- A description of any restrictions on data availability
- For clinical datasets or third party data, please ensure that the statement adheres to our policy

Bulk and single cell RNA sequencing data that support the findings of this study have been deposited in NCBI Sequence Read Archive (SRA) and the Gene Expression Omnibus (GEO) and can be found under GSE216034 (https://www.ncbi.nlm.nih.gov/geo/query/acc.cgi?acc=GSE216034), GSE210556 (https://www.ncbi.nlm.nih.gov/geo/query/acc.cgi?acc=GSE210556) and GSE210557 (https://www.ncbi.nlm.nih.gov/geo/query/acc.cgi?acc=GSE210557). For a subset of transplants, both bulk and scRNAseq is contained in GSE216034. For the remainder of the transplants, bulk RNAseq is contained in GSE210556 and scRNAseq in GSE210557. Molecular Signatures Database (MsigDB) is available at https://www.gsea-msigdb.org/gsea/msigdb/. The KEGG database is available at https://www.genome.jp/kegg/pathway.html. The GO database is available at http://geneontology.org/. ENSEMBL Sscrofa 11.1 and ENSEMBL Macaca fascicularis 6.0 FASTA assemblies are available at https://www.ensembl.org/index.html. Additional data underlying the figures is available in the source data file.

Human research participants

Policy information about studies involving human research participants and Sex and Gender in Research.

Reporting on sex and gender	not applicable
Population characteristics	not applicable
Recruitment	not applicable
Ethics oversight	not applicable

Note that full information on the approval of the study protocol must also be provided in the manuscript.

Field-specific reporting

Please select the one below that is the best fit for your research. If you are not sure, read the appropriate sections before making your selection.

☒ Life sciences ☐ Behavioural & social sciences ☐ Ecological, evolutionary & environmental sciences

For a reference copy of the document with all sections, see nature.com/documents/nr-reporting-summary-flat.pdf

Life sciences study design

All studies must disclose on these points even when the disclosure is negative.

Sample size	No sample-size calculations were performed. The number of transplants reported was based on the total number of kidney xenotransplantations conducted at MGH by the Kawai Lab between November 1, 2020 and May 31, 2022 using eGenesis porcine donors.
Data exclusions	Transplants that occurred within the above time period, but lasted less than 60 days were excluded as the goal of this research was to characterize the impact of xenotransplantation on physiological pathways in stable recipients. For all reported transplants, all data points available at the time the manuscript was drafted were included.
Replication	For the majority of the analyses, 6 or more separate transplants were included. For each sample type in the scRNAseq data set, at least two individuals were included. All replicate data generated has been reported.
Randomization	Recipients were selected for xenotransplantation studies primarily according to their availability at the time of transplants. All recipients included in the main study received a porcine xenograft, therefore randomization was not possible. For the majority of the analyses, the most relevant comparisons were conducted on the level of the indivdual, ie post-xenotransplant compared to pre-xenotransplant.
Blinding	Samples were collected and some analyses were run by the team who conducted the organ procurement and transplantation. Therefore it was not possible to blind the investigators to the recipients status as pre or post transplant.

Reporting for specific materials, systems and methods

We require information from authors about some types of materials, experimental systems and methods used in many studies. Here, indicate whether each material, system or method listed is relevant to your study. If you are not sure if a list item applies to your research, read the appropriate section before selecting a response.

Materials & experimental systems

n/a	Involved in the study
☐	☒ Antibodies
☒	☐ Eukaryotic cell lines
☒	☐ Palaeontology and archaeology
☐	☒ Animals and other organisms
☒	☐ Clinical data
☒	☐ Dual use research of concern

Methods

n/a	Involved in the study
☒	☐ ChIP-seq
☒	☐ Flow cytometry
☒	☐ MRI-based neuroimaging

Antibodies

Antibodies used	anti-CD20 [2B8R1F8]-Afucosylated antibody (NIH Nonhuman Primate Reagent Resource [NIH-NPRR], Cat# PR-8288, RRID:AB_2819341, 20 mg/kg), Anti-rhesus thymocyte(NIH-NPRR, Cat# PR-0000e, RRID:AB_2716327, 5 mg/kg), anti-CD154 mAb [5C8H1] (NIH-NPRR, Cat# PR-1547, RRID:AB_2716324, 25 mg/kg), TNX-1500 [anti-CD154] (Tonix Pharmaceuticals, 25 mg/kg)
Validation	Information about the antibodies from NIH-NPRR are available at their website (https://www.nhpreagents.org/). Additional information as well as citations for publications reporting on TNX-1500 can be found at the Tonix Website (https://www.tonixpharma.com/)

Animals and other research organisms

Policy information about studies involving animals; ARRIVE guidelines recommended for reporting animal research, and Sex and Gender in Research

Laboratory animals	Porcine Donors: Sus scrofa, Yucatan minipigs, intentionally genetically altered, 1.2 - 4.2 months of age, 6.3 - 27 kg<br>NHP Recipients: Macaca fascicularis, estimated age 4.6 - 9.1 years old, 4.4 - 8.7 kg
Wild animals	Some of the NHP recipients used in this study were wild-caught. These animals were obtained from BC US LCC.
Reporting on sex	All porcine donors were female. Recipients included 4 females and 13 males.
Field-collected samples	No field collected samples were used in this study.
Ethics oversight	Massachusetts General Hospital Institutional Animal Care and Use Committee (Protocols 2017N000216 and 2017N000214)

Note that full information on the approval of the study protocol must also be provided in the manuscript.
